# Three-Year Outcome of VBX Stent Graft Used as a Bridging Stent in Endovascular Repair of Post-Dissection Thorachoabdominal Aortic Aneurysm

**DOI:** 10.3390/jcdd13070311

**Published:** 2026-07-06

**Authors:** Frida Jonsdottir, Luca Bertoglio, Timothy Resch

**Affiliations:** 1 Department of Vascular Surgery, Rigshospitalet, 2100 Copenhagen, Denmark; timothy.andrew.resch@regionh.dk; 2 Department of Clinical and Experimental Sciences (DSCS), University and ASST Spedali Civil Hospital of Brescia, 25123 Brescia, Italy

**Keywords:** PD-TAAA, bridging stents, F/B-EVAR, VBX

## Abstract

Post-dissection thoracoabdominal aortic aneurysm (PD-TAAA) is a late sequela of chronic aortic dissection. Complex endovascular aneurysm repair (EVAR), including fenestrated and branched techniques (F/B-EVAR), enables aneurysm exclusion while preserving visceral perfusion; however, bridging stents are not specifically designed for PD-TAAA and are frequently used off-label. Evidence on bridging stent performance is largely derived from degenerative aneurysm cohorts, and PD-TAAA-specific data remain limited. This study evaluated outcomes of the VBX Stent Graft when used as a bridging stent during F/B-EVAR for PD-TAAA. This retrospective analysis included patients with PD-TAAA from the EMBRACE registry (ClinicalTrials.gov: NCT05143138), a multicenter, single-arm registry with retrospective and prospective components, with all outcomes core-laboratory-adjudicated. Procedural, early (thirty-day), and midterm outcomes at one and three years were assessed. The primary endpoints were all-cause mortality and freedom from target vessel instability, defined as loss of durable target vessel reconstruction. Twenty-one patients (mean age 61.5 years; range, 28–77 years) underwent F/B-EVAR with at least one VBX Stent Graft. In total, 82 visceral arteries were treated, of which 51 were bridged with a VBX Stent Graft. Technical success was 100%. Two serious adverse events occurred perioperatively, one requiring reintervention, with no thirty-day mortality or major adverse events. Freedom from all-cause mortality was 95.2% at one year and 90.5% at three years, with two deaths during follow-up. Freedom from target vessel instability at the patient level was 85.7% at both one and three years (95% CI, 62.0–95.2%). VBX Stent Grafts used as bridging stents during F/B-EVAR for PD-TAAA demonstrated high technical success, low early morbidity and mortality, and acceptable mid-term survival and target vessel stability, supporting their use in this challenging anatomical setting within the limitations of a small PD-TAAA cohort.

## 1. Introduction

Post-dissection thoracoabdominal aortic aneurysm (PD-TAAA) is a late sequela of chronic aortic dissection, characterized by progressive aneurysmal degeneration of the thoracoabdominal aorta involving visceral segments [1]. Complex endovascular aortic repair (EVAR), including fenestrated and branched techniques, has emerged as a viable treatment option for PD-TAAA, allowing aneurysm exclusion while preserving visceral perfusion [2]. At present, bridging stents used during fenestrated or branched endovascular repair (F/B-EVAR) of PD-TAAA are not specifically designed for this indication and are used off-label [3]. PD-TAAA differs from degenerative aneurysms in two key anatomic aspects. The true lumen (TL) is commonly reduced in diameter due to false-lumen (FL) compression, and visceral branches may arise from either the true or false lumen [4]. Most of the available evidence regarding the performance of bridging stents in F/B-EVAR originates from studies of degenerative aortic aneurysms. In contrast, data specifically addressing bridging stent outcomes in PD-TAAA remain limited, leaving the optimal bridging stent choice in this setting largely unknown [3]. The GORE^®^ VIABAHN^®^ VBX Balloon Expandable Stent Graft (VBX, W. L. Gore & Associates, Flagstaff, AZ, USA) has a CE mark under the European Medical Device Regulation, indicated for use in F/B-EVAR [5]. However, no device is specifically indicated for bridging stent use in PD-TAAA. The VBX Stent Graft is widely used but limited published evidence exists in PD-TAAA [6].

This study aimed to evaluate the technical success, target vessel patency, and clinical outcomes of the VBX Stent Graft when used as a bridging stent during endovascular repair of PD-TAAA after one and three years.

## 2. Materials and Methods

This study is a retrospective analysis of data derived from the EMBRACE registry (ClinicalTrials.gov: NCT05143138), a multicenter, single-arm registry with retrospective and prospective components designed to evaluate the clinical performance and safety of the VBX Stent Graft when used as a bridging stent during fenestrated and branched endovascular aortic aneurysm repair involving the renal–mesenteric arteries. All outcomes are core laboratory analyzed. The design and methodology of the EMBRACE study have been previously published [7].

For the present analysis, patients with PD-TAAA treated within the EMBRACE registry were identified. Procedural outcomes, early (30-day) outcomes and mid-term outcomes at one and three years were analyzed. Procedural outcomes included technical success and target vessel reconstruction characteristics, while early outcomes included all-cause mortality and major adverse events (MAE) [8]. Mid-term outcomes comprised all-cause mortality, freedom from endoleak, freedom from aneurysm growth, freedom from target vessel instability, target vessel patency, and reintervention. The primary outcomes measured in the present analysis were all-cause mortality and freedom from target vessel instability. Target vessel instability was defined as any loss of durable target vessel reconstruction, including target vessel occlusion, clinically relevant stenosis, loss of alignment or migration, endoleak involving the target vessel, or the need for target vessel–related reintervention [8]. Secondary outcomes included freedom from endoleak, freedom from aneurysm growth, target vessel patency, and reintervention.

### Statistical Analysis

Continuous variables with a normal distribution are presented as mean ± standard deviation, whereas skewed continuous variables are expressed as median with interquartile range. Categorical variables are reported as absolute numbers and percentages, and Kaplan–Meier curves were used to analyze time-dependent outcomes in this study which are presented with standard error. 

Curation of the data and statistical analysis was done by professional statisticians with the use of SAS v9.4.

## 3. Results

### 3.1. Patient Cohort

A total of 21 patients (mean age 61.5 years; range, 28–77 years), including 18 men (85.7%) and 3 women (14.3%), were treated with F/B-EVAR for PD-TAAA and received at least one VBX Stent Graft. Prior aortic intervention was common, with 95% of patients having undergone at least one previous procedure, including thoracic-EVAR (TEVAR) and open repair (each present in 70% of patients).

All baseline characteristics and prior aortic surgeries are listed in Table 1 and Table 2.

### 3.2. Procedural and Early (30-Day) Outcomes

A total of 82 visceral arteries (celiac, superior mesenteric and renal) were treated in the study cohort. Of these, 51 target vessels were reconstructed using a GORE^®^ VIABAHN^®^ VBX Balloon Expandable Stent Graft (VBX, W. L. Gore & Associates, Flagstaff, AZ, USA while the remaining 31 target vessels were treated exclusively with non-VBX covered stents. Unless otherwise specified, outcomes reported are related only to VBX Stent Graft-treated target vessels. Technical success was achieved in all cases (100%). The VBX Stent Graft was used either as a standalone bridging stent or in combination with other devices. Target vessel configuration included 34 fenestrations (41.5%), 45 internal/external branches (54.9%), and 3 internal branches (3.7%); of these, seven target vessels (8.5%) were dissected preoperatively. Detailed target vessel characteristics and reconstruction strategies are summarized in Table 3.

Two serious adverse events occurred at the time of the procedure, consisting of one rupture of the treated segment and one endoleak, the latter of which required reintervention. No intraoperative device failures or procedure-related MAEs were observed. There was no mortality within 30 days of the procedure and no MAEs during the 30-day follow-up period. One adverse event occurred within 30 days that subsequently resulted in death at day 34.

### 3.3. Mid-Term Outcomes at 1 and 3 Years

**Figure 1:**
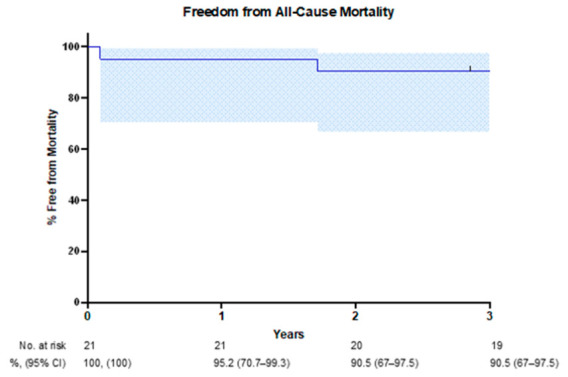
Kaplan–Meier estimate of all-cause mortality over a 3-year follow-up period. Tick marks indicate censored observations. The shaded area represents the 95% confidence interval. Numbers at risk are shown below the x-axis at the beginning of each time interval, along with corresponding estimations and 95% confidence intervals.

Clinical follow-up was complete for all patients. Computed tomography (CT) imaging was available in 100% of patients at one year, 95% at two years, and 94.7% at three years. Freedom from all-cause mortality was 95.2% at 1 year and 90.5% at 3 years, as shown in Figure 1. 

Two deaths occurred during follow-up. One death followed an adverse event that occurred within 30 days of the procedure and resulted in death at day 34; this death was adjudicated as aneurysm-related and procedure-related and was caused by a right renal hematoma. A second death occurred during the second year of follow-up and was not aneurysm related.

**Figure 2:**
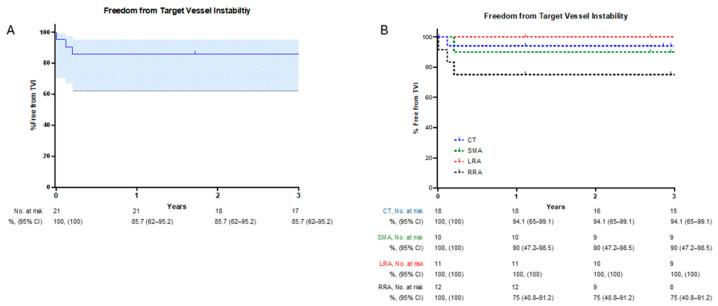
Freedom from target vessel instability. (**A**) Subject-level Kaplan–Meier estimates of Freedom from target vessel instability (TVI) over 3 years. Tick marks denote censored observations. The shaded region represents the 95% confidence interval. Numbers at risk, rate estimates, and 95% confidence intervals are provided below the x-axis. (**B**) Kaplan–Meier estimates of Freedom from target vessel instability stratified by target vessel over the 3-year follow-up period. Tick marks indicate censoring. Numbers at risk, rate estimates, and 95% confidence intervals are provided below the x-axis. CT = celiac trunk; SMA = superior mesenteric artery; LRA = left renal artery; RRA = right renal artery.

At the patient level, freedom from target vessel instability was 85.7% at both one and three years (95% CI, 62.0–95.2%). At the vessel level among VBX Stent Graft-treated target vessels, no instability was observed in left renal arteries. Freedom from instability of the right renal artery was 75% at both one and three years, while freedom from target vessel instability at one and three years was 90% for the superior mesenteric artery and 94.1% for the celiac trunk. Freedom from target vessel instability is shown in Figure 2.

**Figure 3:**
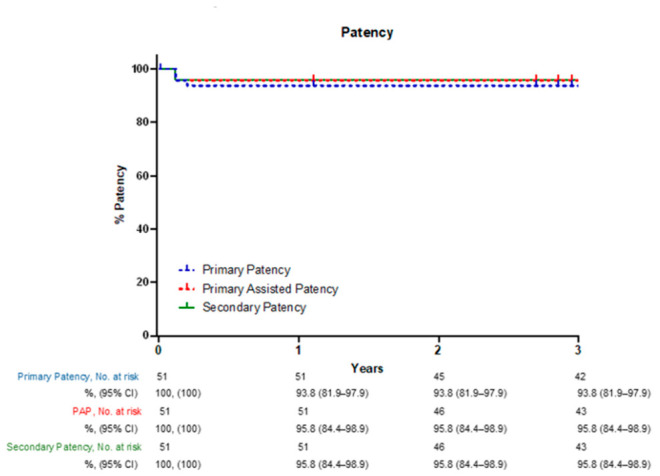
Vessel-level patency. Kaplan–Meier estimates of vessel-level primary patency, primary assisted patency, and secondary patency over a 3-year follow-up period. Tick marks indicate censored observations. Numbers at risk, estimate rates, and corresponding 95% confidence intervals are shown below the x-axis. PAP = primary assisted patency.

Target vessel patency estimates in VBX Stent Graft-treated vessels were identical at one and three years. Primary patency was 93.8% (95% CI, 81.9–97.9%), while both primary assisted patency and secondary patency were 95.8% (95% CI, 84.4–98.9%) at the vessel level (see Figure 3). When analyzed by individual vessel, primary and secondary patency at one and three years were 94.1% for the celiac trunk, 90.0% for the superior mesenteric artery, 90.9% for the right renal artery, and 100% for the left renal artery.

By one year, eight reinterventions had been performed overall, including treatment of two stenoses, four endoleaks, one occlusion, and one for other reasons. Three of these reinterventions involved VBX Stent Graft-treated target vessels. Two of the twelve right renal arteries required reinterventions within the first year due to type IC endoleak and were successfully treated endovascularly. Of the ten superior mesenteric arteries treated with a VBX Stent Graft, one required reintervention within the first year because of stenosis and was treated with balloon angioplasty. During the second year of follow-up, one additional reintervention for endoleak was performed that was not related to VBX Stent Graft-treated target vessel. No further reinterventions were recorded at three years.

Freedom from all-cause reintervention at the patient level was 80.2% (95% CI, 55.4–92.1%) at both one and three years. At one year, freedom from reintervention among VBX Stent Graft-treated target vessels was 90.2% (95% CI, 66.2–97.5%) at the patient level. Freedom from endoleak in VBX Stent Graft-treated target vessels was 95.8% (95% CI, 84.4–98.9%) at both one and three years. Freedom from aneurysm growth was 94.4% (95% CI; 66.6–99.2%) at one year and decreased to 77% (95% CI, 49.7–90.7%) at three years.

## 4. Discussion

The present study evaluated procedural, early, and mid-term outcomes of VBX Stent Grafts used as bridging stents during F/B-EVAR for PD-TAAA. The main findings were a technical success rate of 100% with no 30-day mortality. At mid-term follow-up, freedom from all-cause mortality was 95.2% at one year and 90.5% at three years, and freedom from target vessel instability was 85.7% at both time points.

The present findings are broadly in line with previously published data on the use of VBX Stent Grafts as bridging stents in F/B-EVAR. Kappe et al. reported technical success rates of 97.5% and favorable mid-term durability, including a freedom from target vessel instability of 97.8% at one year in a predominantly degenerative aneurysm cohort [9]. Similarly, results from the EXPAND registry reported by Usai et al. demonstrated favorable mid-term outcomes, with freedom from target vessel instability at 94.5% at three years [10]. In the present study, technical success was achieved in all cases (100%), however, freedom form target vessel instability at one year was lower at 85.7%. This difference may reflect the increased anatomical complexity of PD-TAAA, including TL compression and variable vessel origin, as well as the limited sample size of the present cohort, as indicated by the wide confidence intervals.

At the vessel level, Usai et al. reported primary and secondary patency rates of 93.6% (95% CI, 88.9–96.3%) and 96.8% (95% CI, 93.1–98.6%), respectively. In comparison, primary patency in the present study was similar at 93.8%, while secondary patency was slightly lower at 95.8% at both one and three years.

In contrast, outcomes of F/B-EVAR specifically in PD-TAAA have been reported by Gorgatti et al., who demonstrated high technical success (96%) but also substantial perioperative risk, including a thirty-day mortality rate of 7% and major adverse events in approximately 20% of patients, as well as freedom from reintervention of 55% at two years [11]. In the present study, no thirty-day mortality or major adverse events were observed, and freedom from reintervention was 80.2% at both one and three years, suggesting lower observed reintervention rates in the present cohort. These differences may reflect variations in patient selection, procedural strategies, and device use; however, they suggest that favorable outcomes can be achieved despite the inherent anatomical complexity of PD-TAAA.

Importantly, while the studies by Kappe et al. and Usai et al. primarily included patients with degenerative aneurysms, the present analysis focuses exclusively on PD-TAAA. Despite the challenges associated with a compressed TL and complex visceral vessel anatomy, the observed outcomes remained comparable to those reported in mixed or degenerative cohorts, supporting the feasibility and mid-term durability of VBX Stent Grafts in this high-risk population.

The observed difference in target vessel instability between renal arteries may be partly explained by the anatomical characteristics of aortic dissection. In PD-TAAA, renal arteries may originate from TL, FL, or both, and true lumen compression can further complicate target vessel reconstruction. Previous studies have demonstrated that renal arteries affected by dissection, including those supplied by the FL or both lumens, are more frequently located on the right side, whereas left renal arteries are more commonly supplied exclusively by the TL [12]. As a result, bridging stent implantation in the right renal artery may be more susceptible to suboptimal expansion, malalignment, or long-term instability compared with the left renal artery. The lower overall freedom from target vessel instability observed in the present study may, in part, be influenced by the reduced stability in right renal arteries, whereas no instability was observed in left renal arteries. However, these findings should be interpreted with caution given the limited number of vessel-specific events.

## 5. Limitations

This study has several limitations. First, it represents a retrospective, exploratory subset analysis of registry data, which is subject to inherent selection bias. Second, the sample size is relatively small, limiting the statistical power and generalizability of the findings. Third, no comparative analysis with other bridging stent devices was performed. In addition, the limited sample size precluded meaningful subgroup analyses according to target vessel origin from the true or false lumen, which may influence bridging stent performance in PD-TAAA. Finally, vessel-specific subgroup analyses were based on small numbers and estimates of target vessel instability should therefore be interpreted with caution.

## 6. Conclusions

VBX Stent Grafts used as bridging stents during F/B-EVAR for PD-TAAA demonstrated high technical success, low early morbidity and mortality, and acceptable mid-term survival and target vessel stability in the present analysis. These findings suggest that VBX Stent Grafts may provide a feasible and durable option for target vessel reconstruction in these challenging anatomical settings. Further studies with larger cohorts and longer follow-up are warranted to confirm these results.

## Figures and Tables

**Table 1:** Baseline Characteristics and Medical History.

Number of Subjects	21
Gender	
Male	18 (85.7%)
Female	3 (14.3%)
Age (years)	
Mean (Std Dev)	61.5 (14.1)
Median	66
(Min, Max)	(28, 77)
Anticoagulant Prior To Procedure *	8 (40.0%)
Anticoagulant Type	
Vitamin K antagonist	7 (87.5%)
Direct oral anticoagulant	1 (12.5%)
Smoking (Current/Former)	11 (52.4%)
Chronic Kidney Disease	
None	18 (85.7%)
Stage 1–2	1 (4.8%)
Stage 3–4	2 (9.5%)
Stage 5	0 (0%)
Hypercholesterolemia	9 (42.9%)
Diabetes	0 (0%)
Chronic obstructive pulmonary disease	3 (14.3%)
Other Lung Disease	1 (4.8%)
Myocardial Infarction	2 (9.5%)
Congestive Heart Failure	5 (23.8%)
Valvular Heart Disease	5 (23.8%)
Cardiac Arrhythmia	4 (19.0%)
Paraplegia	1 (4.8%)
Cancer (resolved)	2 (9.5%)
Renal Dialysis	0 (0%)
Connective Tissue Disorder	7 (33.3%)
Stroke	1 (4.8%)
Other Vascular Intervention	10 (47.6%)

* *n* = 20; Percentages are calculated among patients with available data; missing values were excluded. Values are presented as *n* (%) or mean (standard deviation).

**Table 2:** Prior Aortic Surgeries.

Number of Subjects Enrolled	21
Previous Aortic Surgeries	20 (95.2%)
Number Of Previous Aortic Surgeries	
1	8 (40.0%)
2	6 (30.0%)
3	4 (20.0%)
4 or more	2 (10.0%)
Type Of Previous Aortic Surgery (more than one can apply)	
Open Repair	14 (70.0%)
tEVAR	14 (70.0%)
EVAR	1 (5.0%)
bEVAR	1 (5.0%)
Pathology Treated (more than one can apply)	
Type A Dissection	4 (20.0%)
Type B Dissection	14 (70.0%)
Ascending Aortic Aneurysm	3 (15.0%)
Descending Thoracic Aortic Aneurysm	6 (30.0%)
Thoracoabdominal Aortic Aneurysm	5 (25.0%)
Abdominal Aortic Aneurysm	4 (20.0%)
Other	5 (25.0%)
Combinations of different pathology options reported in the data	
Abdominal Aortic Aneurysm	3 (15.0%)
Ascending Aortic Aneurysm	1 (5.0%)
Ascending Aortic Aneurysm, Descending Thoracic Aortic Aneurysm	1 (5.0%)
Descending Thoracic Aortic Aneurysm	2 (10.0%)
Thoracoabdominal Aortic Aneurysm	4 (20.0%)
Thoracoabdominal Aortic Aneurysm, Abdominal Aortic Aneurysm	1 (5.0%)
Type A Dissection	3 (15.0%)
Type A Dissection, Type B Dissection, Ascending Aortic Aneurysm, Descending Thoracic Aortic Aneurysm	1 (5.0%)
Type A Dissection, Type B Dissection, Thoracoabdominal Aortic Aneurysm	1 (5.0%)
Type B Dissection	12 (60.0%)
Type B Dissection, Descending Thoracic Aortic Aneurysm	2 (10.0%)

Percentages are calculated among patients with available data; missing values were excluded. Values are presented as *n* (%). tEVAR: Thoracal Endovascular aneurysm repair; EVAR: Endovascular aneurysm repair; bEVAR: Branched Endovascular aneurysm repair.

**Table 3:** Target vessel characteristics and reconstruction details.

	Overall	Celiac Trunk	SMA	Left Renal	Right Renal
Arteries Treated	82	20	21	21	20
Status of Artery Prior To Procedure					
Patent	81 (98.8%)	19 (95.0%)	21 (100.0%)	21 (100.0%)	20 (100.0%)
Stenosis > 50%	0 (0%)	0 (0%)	0 (0%)	0 (0%)	0 (0%)
25% < Stenosis ≤ 50%	1 (1.2%)	1 (5.0%)	0 (0%)	0 (0%)	0 (0%)
≤25% Stenosis	0 (0%)	0 (0%)	0 (0%)	0 (0%)	0 (0%)
Vessel Dissected	7 (8.5%)	1 (5.0%)	1 (4.8%)	3 (14.3%)	2 (10.0%)
Artery Diameter at Landing Zone (n)	64	16	16	17	15
Mean (Std Dev)	8.1 (2.2)	10.3 (1.9)	9.0 (1.6)	6.6 (1.4)	6.5 (1.2)
Median	8.0	10.5	9.0	6.0	6.0
(Min, Max)	(4.4, 13.0)	(7.0, 13.0)	(5.5, 12.0)	(4.4, 9.0)	(4.5, 9.0)
Portal Configuration					
Fenestration	34 (41.5%)	5 (25.0%)	7 (33.3%)	12 (57.1%)	10 (50.0%)
External branch	45 (54.9%)	14 (70.0%)	12 (57.1%)	9 (42.9%)	10 (50.0%)
Internal branch	3 (3.7%)	1 (5.0%)	2 (9.5%)	0 (0%)	0 (0%)
Number Of Devices Implanted					
1	62 (75.6%)	14 (70.0%)	17 (81.0%)	16 (76.2%)	15 (75.0%)
2	14 (17.1%)	3 (15.0%)	4 (19.0%)	4 (19.0%)	3 (15.0%)
3	4 (4.9%)	1 (5.0%)	0 (0%)	1 (4.8%)	2 (10.0%)
More than 3	2 (2.4%)	2 (10.0%)	0 (0%)	0 (0%)	0 (0%)
VBX Stent Graft Implanted					
VBX Stent Graft Only	38 (46.3%)	12 (60.0%)	9 (42.9%)	8 (38.1%)	9 (45.0%)
VBX Stent Graft Concomitantly	13 (15.9%)	6 (30.0%)	1 (4.8%)	3 (14.3%)	3 (15.0%)
No VBX Stent Graft	31 (37.8%)	2 (10.0%)	11 (52.4%)	10 (47.6%)	8 (40.0%)
Devices Implanted	82	20	21	21	20
VBX Stent Graft	38 (46.3%)	12 (60.0%)	9 (42.9%)	8 (38.1%)	9 (45.0%)
Gore Viabahn (self-expanding)	1 (1.2%)	0 (0%)	0 (0%)	0 (0%)	1 (5.0%)
Advanta V12	10 (12.2%)	1 (5.0%)	4 (19.0%)	3 (14.3%)	2 (10.0%)
Bard Covera	2 (2.4%)	0 (0%)	1 (4.8%)	1 (4.8%)	0 (0%)
Bentley BeGraft	9 (11.0%)	0 (0%)	2 (9.5%)	4 (19.0%)	3 (15.0%)
Bentley BeGraft Plus	1 (1.2%)	0 (0%)	1 (4.8%)	0 (0%)	0 (0%)
Other	3 (3.7%)	1 (5.0%)	1 (4.8%)	0 (0%)	1 (5.0%)
VBX Stent Graft, Gore Viabahn (self expanding)	2 (2.4%)	0 (0%)	0 (0%)	1 (4.8%)	1 (5.0%)
VBX Stent Graft, Gore Viabahn (self expanding), Bare Metal Stent	1 (1.2%)	0 (0%)	0 (0%)	0 (0%)	1 (5.0%)
VBX Stent Graft, Advanta V12, Bare Metal Stent	1 (1.2%)	1 (5.0%)	0 (0%)	0 (0%)	0 (0%)
VBX Stent Graft, Bard Fluency, Bare Metal Stent	1 (1.2%)	1 (5.0%)	0 (0%)	0 (0%)	0 (0%)
VBX Stent Graft, Bentley BeGraft	1 (1.2%)	0 (0%)	0 (0%)	1 (4.8%)	0 (0%)
VBX Stent Graft, Bentley BeGraft, Bare Metal Stent	1 (1.2%)	1 (5.0%)	0 (0%)	0 (0%)	0 (0%)
VBX Stent Graft, Bentley BeGraft, Other	1 (1.2%)	0 (0%)	0 (0%)	1 (4.8%)	0 (0%)
VBX Stent Graft, Bentley BeGraft Plus	1 (1.2%)	1 (5.0%)	0 (0%)	0 (0%)	0 (0%)
VBX Stent Graft, Bare Metal Stent	4 (4.9%)	2 (10.0%)	1 (4.8%)	0 (0%)	1 (5.0%)

Percentages are calculated among target vessels with available data; missing values were excluded. Values are presented as *n* (%). SMA: Superior mesenteric artery.

## Data Availability

The datasets presented in this article are not readily available because the data are part of an ongoing study. Requests to access the datasets should be directed to W.L. Gore & Associates.

## Abbreviations

The following abbreviations are used in this manuscript:

PD-TAAA	Post-dissection thoracoabdominal aortic aneurysm
EVAR	Endovascular aneurysm repair
F/B-EVAR	Fenestrated and branched endovascular aneurysm repair
TL	True lumen
FL	False lumen
SMA	Superior mesenteric artery
TEVAR	Thoracal-EVAR
CT	Computed tomography
VBX	GORE^®^ VIABAHN^®^ VBX Balloon Expandable Stent Graft
